# Alterations in Genome Organization in Lymphoma Cell Nuclei due to the Presence of the t(14;18) Translocation

**DOI:** 10.3390/ijms25042377

**Published:** 2024-02-17

**Authors:** Elisa Garimberti, Concetta Federico, Denise Ragusa, Francesca Bruno, Salvatore Saccone, Joanna Mary Bridger, Sabrina Tosi

**Affiliations:** 1 Clinical Genomics Laboratory, Royal Marsden NHS Foundation Trust, London SW3 6JJ, UK; elisa.garimberti@rmh.nhs.uk; 2 Department of Biological, Geological, and Environmental Sciences, University of Catania, Via Androne 81, 95124 Catania, Italy; federico@unict.it (C.F.); francesca.bruno@unict.it (F.B.); saccosal@unict.it (S.S.); 3 Centre for Genome Engineering and Maintenance (CenGEM), College of Health, Medicine and Life Sciences, Brunel University London, Kingston Lane, Uxbridge UB8 3PH, UK; denise.ragusa@brunel.ac.uk (D.R.); joanna.bridger@brunel.ac.uk (J.M.B.); 4 Leukaemia and Chromosome Research Laboratory, College of Health, Medicine and Life Sciences, Brunel University London, Kingston Lane, Uxbridge UB8 3PH, UK

**Keywords:** chromosomes, chromosomal translocation, t(14;18), *BCL2*, *IGH*, genome organization, nuclear gene positioning, lymphoma, chromosome looping, fluorescence in situ hybridization

## Abstract

Chromosomal rearrangements have been shown to alter genome organization, consequently having an impact on gene expression. Studies on certain types of leukemia have shown that gene expression can be exacerbated by the altered nuclear positioning of fusion genes arising from chromosomal translocations. However, studies on lymphoma have been, so far, very limited. The scope of this study was to explore genome organization in lymphoma cells carrying the t(14;18)(q32;q21) rearrangement known to results in over-expression of the *BCL2* gene. In order to achieve this aim, we used fluorescence in situ hybridization to carefully map the positioning of whole chromosome territories and individual genes involved in translocation in the lymphoma-derived cell line Pfeiffer. Our data show that, although there is no obvious alteration in the positioning of the whole chromosome territories, the translocated genes may take the nuclear positioning of either of the wild-type genes. Furthermore, the *BCL2* gene was looping out in a proportion of nuclei with the t(14;18) translocation but not in control nuclei without the translocation, indicating that chromosome looping may be an essential mechanism for *BCL2* expression in lymphoma cells.

## 1. Introduction

Follicular lymphoma is a notoriously indolent type of non-Hodgkin lymphoma affecting B-cell lineage in adults. A classical hallmark of follicular lymphoma is the chromosomal translocation t(14;18)(q32;q21), which is present in 85–90% of cases [1,2,3]. The mechanisms of tumorigenesis associated with t(14;18) have been attributed to a block of apoptosis brought about by an ectopic over-expression of the anti-apoptotic factor *BCL2*. The translocation, in fact, relocates the *BCL2* gene within the transcriptional influence of the IGH enhancer [4]. Additional cooperating events are, however, required for full transformation and disease manifestation [5,6,7,8].

It is now well established that chromosomal rearrangements can disturb the arrangement of the genome within the nucleus [9,10,11]. From the observation of non-random occupancy of chromosomes into territories and the transcriptionally dependent localization of individual genes [12,13,14], an increasing number of studies are focusing on the link between nuclear disturbance and the development of disease [9,15,16]. Studies have proven that the localization of genes within the nucleus is generally associated with their transcriptional activity, with the inner portion of the nucleus usually being transcriptionally active and peripheral regions tending to be transcriptionally repressed [17,18,19,20]. It has also been shown that this genomic property is evolutionary conserved in all primates [21].

While various studies have found an association between chromosomal rearrangements and a dysregulation of the genes implicated in leukemia in relation to nuclear organization [22,23], the lymphoma-associated translocation t(14;18) has not been investigated in this context to date.

In this study, we sought to characterize the nuclear localization of chromosomal territories and individual genes involved in the t(14;18) translocation in the Pfeiffer cell line. By using different combinations of FISH probes specific to the loci involved in this translocation, we were able to investigate their functional organization in interphase nuclei of the Pfeiffer cell line and in healthy lymphocytes.

## 2. Results

### 2.1. t(14;18) Translocation and BCL2 Expression in the Pfeiffer Cell Line

**Figure 1:**
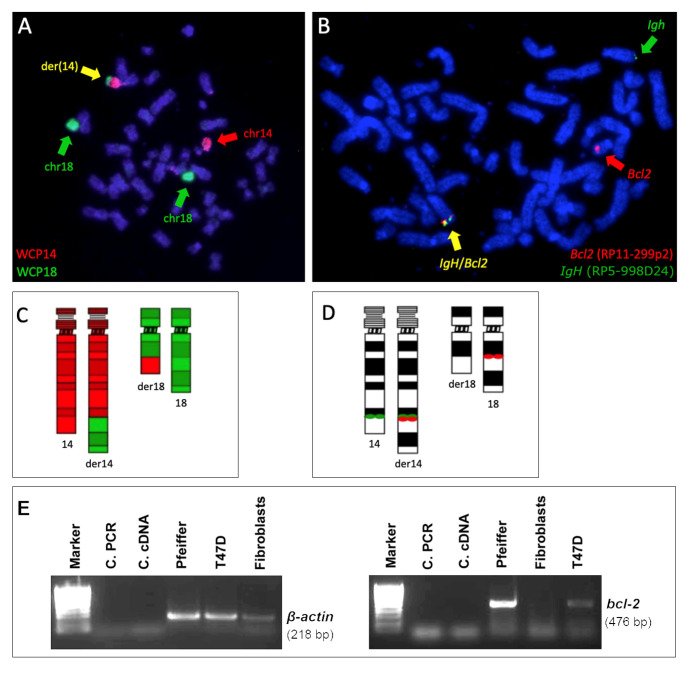
**Visualization of t(14;18) in metaphase chromosomes of the Pfeiffer cell line**. Fluorescence in situ hybridization (FISH) using WCP14 (red), indicated by a red arrow, and WCP18 (green), indicated by a green arrow, probes in a dual-color combination highlights the presence of der(14), indicated by a yellow arrow (**A**). BAC/PAC probes containing *BCL2* (red), indicated by a red arrow, and *IGH* (green), indicated by a green arrow, signals highlight the presence of *IGH:BCL2* gene fusion, indicated by a yellow arrow (**B**). Ideograms of chromosomes 14 and 18 and their respective derivatives provide a schematic representation of the distribution of the painting probes WCP14 (red) and WCP18 (green) (**C**) and of the single-locus probes. The red spots indicate the hybridization in the two chromatids of the chromosome (**D**). Reverse transcription PCR (RT-PCR) showing the presence of a *BCL2* transcript in the Pfeiffer cell line ((**E**), **right panel**). RT-PCR to detect β-actin expression, as a reference control, is shown ((**E**), **left panel**), marker: Hyperladder IV; C.PCR: PCR negative control; C.cDNA: cDNA negative control; T47D: *BCL2* positive control cells; and fibroblasts: *BCL2* negative control cells.

**Figure 2:**
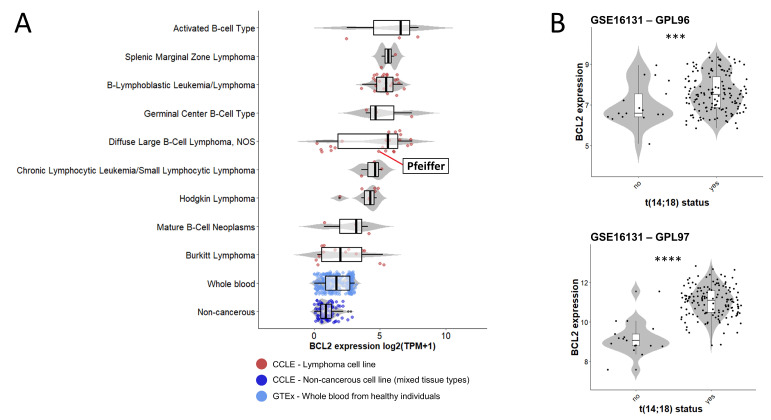
***BCL2* gene expression in lymphoma cell lines and patients**. *BCL2* expression range in different lymphoma cell lines from the Cancer Cell Line Encyclopedia (CCLE), highlighting the Pfeiffer cell line. Expression of BCL2 is also shown in non-cancerous cell lines of various tissue types (CCLE) and in whole blood from healthy individuals (GTEx database). Expression values are retrieved from RNA sequencing in the units of log2(TPM + 1). Boxplots and violin plots summarize data distribution with dots representing individual cell lines or samples, which are color-coded by sample type (**A**). *BCL2* expression in RNA units in patients with diffuse lymphoma with and without t(14;18) from two microarray experiments (GPL96 and GPL97) downloaded from GSE16131. Boxplots and violin plots summarize data distribution with dots representing individual patient samples. Statistical significance is calculated by T test with *p* values ≤ 0.0001 (***), and 0.00001 (****) (**B**).

The presence of t(14;18) in the Pfeiffer cell line was confirmed by FISH using the probe combinations described in the Section 4. Due to the limited extent of the translocated portion from chromosome 14, der(18) was undetectable with the use of whole chromosome painting (WCP) (Figure 1A). The use of both WCP and bacterial artificial clone (BAC) probes, respectively, enabled us to clearly identify der(14) and *IGH::BCL2* gene fusion (Figure 1). The presence of this translocation is consistent with the *BCL2* gene expression detected in this cell line as well as in patients with lymphoma (Figure 2). RNA-seq data from lymphoma cell lines retrieved from the Cancer Cell Line Encyclopedia (CCLE) showed different levels of *BCL2* expression, with the highest levels being observed in diffuse large B-cell lymphoma lines, including the Pfeiffer line, whereas the lowest expression levels were seen in whole blood and non-cancerous tissue (Figure 2A). *BCL2* expression in the Pfeiffer cell lines was also confirmed in-house using reverse transcription (RT) PCR (Figure 1E). We also interrogated the expression of *BCL2* from patients with follicular lymphoma with and without t(14;18) from publicly available data (GSE16131) [24]. *BCL2* expression was significantly higher in patients harboring the translocation compared to patients without t(14;18) in both of the microarray experiments considered in this study (GPL96 and GPL97) (Figure 2B).

### 2.2. Chromosome Territories 14 and 18 Are Unaltered in the Pfeiffer Cell Line Compared to Healthy Lymphocytes

**Figure 3:**
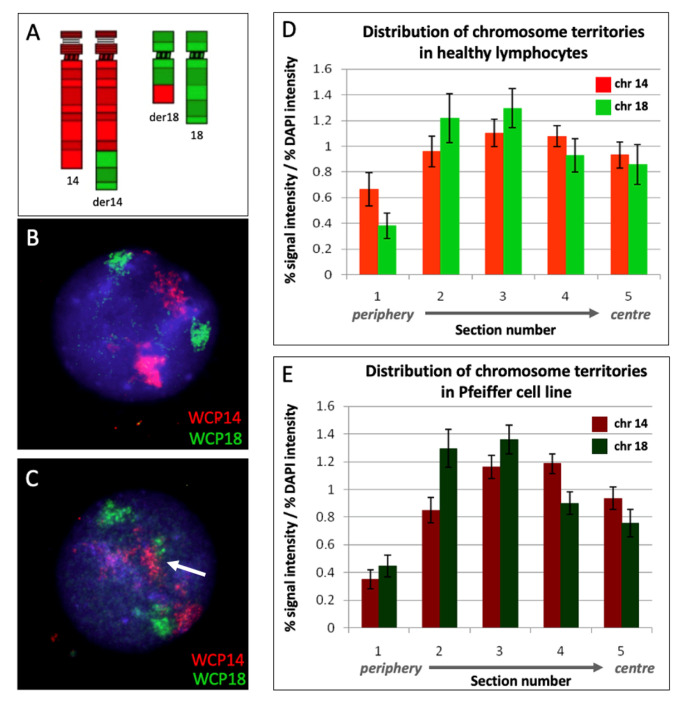
**Distribution of territories of chromosomes 14 and 18 within interphase nuclei of Pfeiffer cells and healthy lymphocytes**. Combination of whole chromosome paint FISH probes for chromosomes 14 (red) and 18 (green) (**A**); example of lymphocyte interphase nucleus used as a control showing distinct territories for chromosomes 14 and 18, respectively (**B**); example of Pfeiffer interphase nucleus showing the fusion of the chromosome territories in der(14) indicated by the white arrow (**C**); erosion analysis of the distribution of chromosome territories in lymphocytes from healthy donors (**D**); and erosion analysis of the distribution of chromosome territories in the Pfeiffer cell line (**E**). The bars are the mean of more than 50 nuclear images analyzed with the erosion script. The error bars are the standard error of the mean. Chromosome 14 and chromosome 18 did not show statistically significant differences in their nuclear location in the Pfeiffer cells compared to the controls (*p* > 0.05).

The distribution of the territories of chromosomes 14 and 18 were determined in nuclei from healthy lymphocytes and the Pfeiffer cell line. When using WCP probes for chromosomes 14 and 18 (Figure 3A), normal lymphocytes revealed an intermediate positioning of chromosome 14 and a peripheral positioning of chromosome 18 (Figure 3B).

The same probe combination was used in the Pfeiffer cell line (Figure 3C). The analysis of the non-translocated chromosomes 14 and 18 did not detect positional shifts in the location of these chromosomes compared to the controls. Specifically, the intermediate positioning of chromosome 14 and the peripheral positioning of chromosome 18 observed in the control lymphocytes (Figure 3D) was maintained in the Pfeiffer cell nuclei (Figure 3E).

### 2.3. Nuclear Positioning of BCL2, IGH, and BCL2::IGH

**Figure 4:**
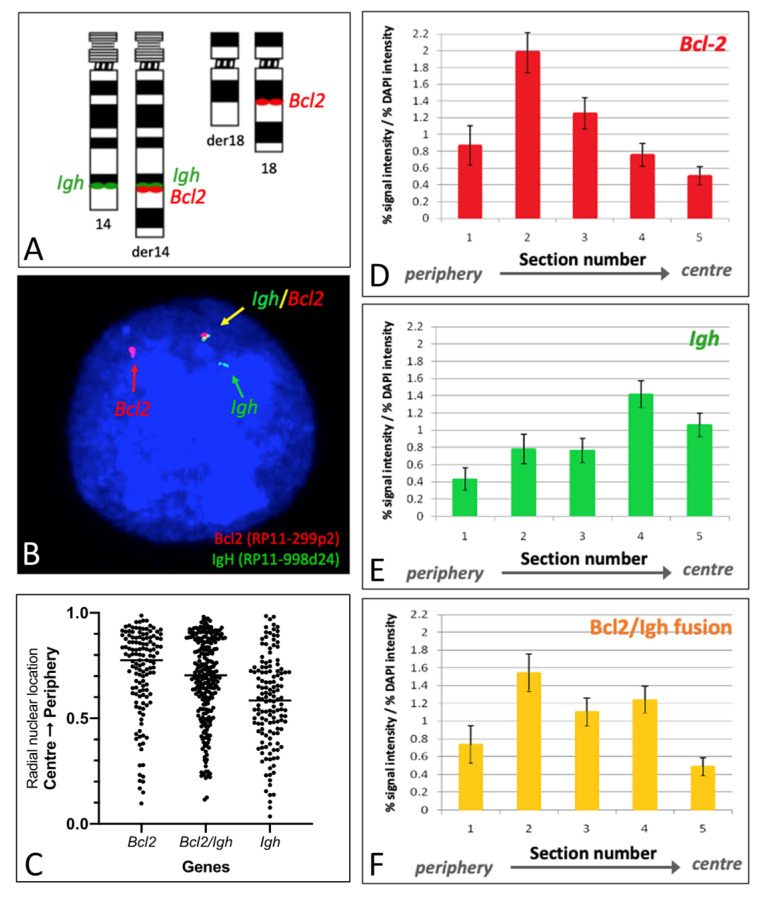
**Nuclear localization of *BCL2* and IGH genes in Pfeiffer nuclei.** Ideogram to show chromosomal locations of the FISH probes used for the *BCL2* (red dots) and IGH (green dots) loci (**A**). Pfeiffer cell line nucleus hybridized with the probe combination shown in panel A, which allows the visualization of the non-translocated *BCL2* in red (indicated by a red arrow), the non-translocated *IGH* in green (indicated by a green arrow), and the fusion signal, red and green close to each other, corresponding to the *BCL2::IGH* fusion (indicated by a yellow arrow) (**B**). Radial nuclear positioning, obtained by a statistical analysis distribution of a hundred signals, shows localization of the wild-type *BCL2* allele towards the periphery of the nucleus, whereas the positioning of the wild-type *IGH* allele is more central, and the fusion *BCL2::IGH* shows a bimodal distribution within two main areas, in the periphery and closer to the center of the nucleus, respectively (**C**). The erosion analysis shows the positioning of the wild-type *BCL2* allele towards the nuclear periphery, mainly in zone 2 (**D**), positioning of the wild-type *IGH* allele towards the inner/intermediate portion of the nucleus, mainly in zone 4–5 (**E**), and a fusion signal corresponding to the *BCL2::IGH* fusion occupying prevalently zone 2 and zone 4, mimicking the positioning of both wild-type alleles (**F**). The bars are the mean of more than 50 nuclear images analyzed with the erosion script. The error bars are the standard error of the mean.

We determined the position of the individual genes involved in t(14;18), namely, *BCL2* (18q21) and *IGH* (14q32), and the resulting fusion gene, *BCL2::IGH*. FISH, using single-locus probes (Figure 4A), allowed the discrimination of the translocated and non-translocated alleles. As shown in a representative nucleus of the Pfeiffer cell line (Figure 4B), the non-translocated *BCL2* locus is visible in red, the non-translocated *IGH* in green, and the fusion on der(14) is detectable in yellow. The positioning of the individual genes within the nucleus was determined by radial nuclear location (RNL) [22] (Figure 4C) and by an erosion analysis based on the evaluation of signal intensity in each nuclear section [25] (Figure 4D,E).

Using both RNL and erosion analysis, the highest signal intensity for the non-translocated *BCL2* allele was found within nuclear zone 2, indicating a preferentially peripheral localization (Figure 4C,D). The non-translocated *IGH* allele displayed an inner/intermediate localization, with a peak in the intermediate nuclear Section 4 (Figure 4C,E). The localization of the *BCL2::IGH* fusion signal presented a bimodal distribution within the analyzed nuclei, with two peaks at sections 2 (more peripheral) and 4 (more internal) mimicking the positioning of both wild-type alleles (Figure 4C,F).

### 2.4. Gene Nuclear Positioning in Relation to Their Corresponding Chromosomal Territories

**Figure 5:**
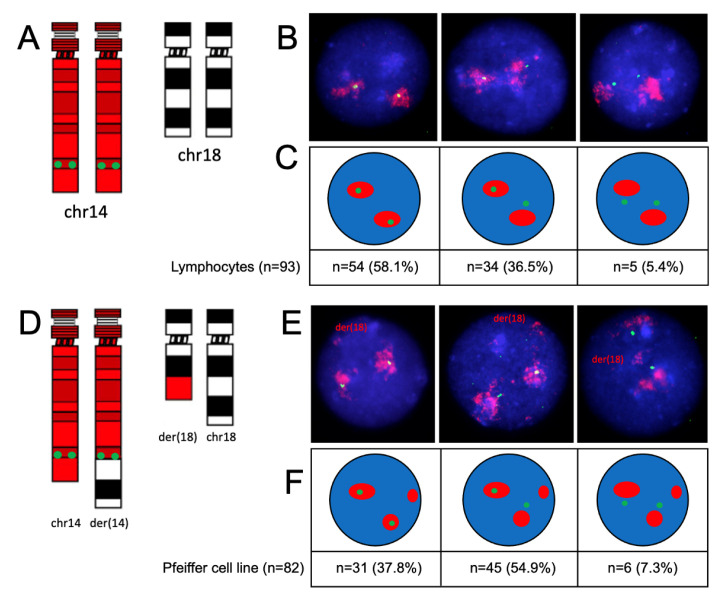
**Nuclear localization of the *IGH* locus in relation to the chromosome 14 territory**. Ideograms showing the probe combination depicts 14WCP in red and the *IGH* probe in green in lymphocytes and Pfeiffer cells ((**A**,**D**), respectively). Examples of lymphocyte nuclei showing WCP14/*IGH* hybridization in lymphocytes and Pfeiffer cells. In the Pfeiffer nuclei der(18) is indicated. Der(14) is not indicated due to being not distinguishable from the normal chromosome 14 ((**B**,**E**), respectively). Schematic representations of the FISH patterns observed, with the different proportions of nuclei with signals, from left to right, specific for both alleles within the chromosome territory, one allele within the chromosome territory, or both alleles looping out of the chromosome territory ((**C**,**F**), respectively).

**Figure 6:**
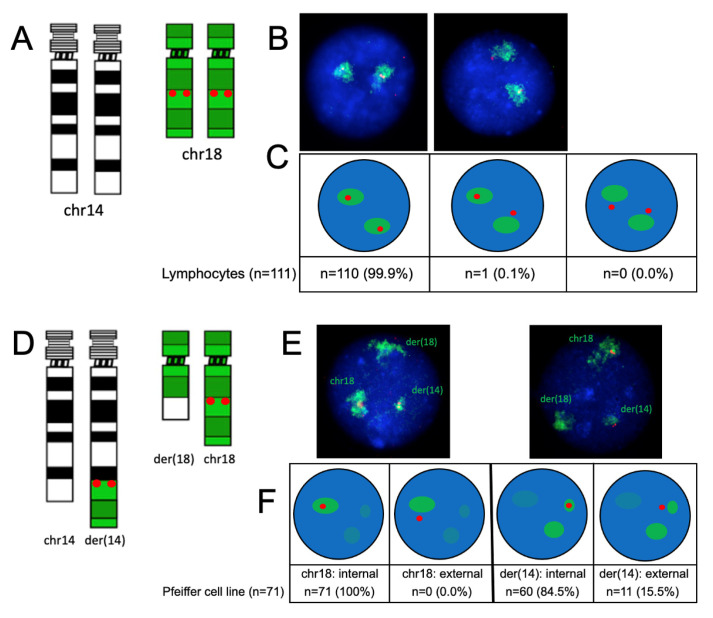
**Nuclear localization of *BCL2* in relation to the chromosome 18 territory**. Ideogram showing the probe combination depicts 18WCP in green and the *BCL2* probe in red in lymphocytes and Pfeiffer cells ((**A**,**D**), respectively). Example of nuclei showing WCP14/*BCL2* hybridization in lymphocytes and Pfeiffer cells. In the Pfeiffer nuclei, the normal chromosome 18 (green territory with red signal), der(14) (smaller green territory with red signals), and der(18) (slightly larger green territory with no red signals) ((**B**,**E**), respectively) are indicated. Schematic representations of the FISH patterns observed in healthy lymphocytes showing different proportions of nuclei with signals specific for both alleles within the chromosome territory, one allele within the chromosome territory, or both alleles looping out of the chromosome territory (**C**); and in Pfeiffer cells showing all *BCL2* wild-type alleles localized within the normal chromosome18 territory and the translocated *BCL2* allele looping out of the der(14) territory in approximately 15% of nuclei (**F**).

In order to define the nuclear localization of individual genes in relation to their respective chromosomal territories, we conducted dual-color FISH by combining WCP paints and single-locus probes for *BCL2* and *IGH*, respectively (Figure 5 and Figure 6). FISH using the *IGH* + 14WCP probe combination showed that the *IGH* gene was localized within the chromosome 14 territory in the majority of lymphocyte nuclei (58.1%), whereas it was looping out in the majority of nuclei in the Pfeiffer cell line from at least one chromosome (54.9% of nuclei) or from both chromosomes (7.3%). Looping out of the *IGH* locus in the Pfeiffer cell line is statistically different compared to the control cells (*p* = 0.005 evaluated by a Chi-square test) (Figure 5C). FISH using the *BCL2* + 18WCP probe combination showed that almost 100% of lymphocyte nuclei retained the *BCL2* signal within the chromosome 18 territory, whereas, in the Pfeiffer cell line, the *BCL2* signal was found within the chromosome 18 territory when observing the non-translocated chromosome 18 and was looping out of the der(14) territory in 15.5% of the nuclei. Looping out of the *BCL2* locus in the Pfeiffer cell line is significantly statistically different compared to the control cells (*p* = 0.005 evaluated by Chi-square test) (Figure 6C).

## 3. Discussion

The Pfeiffer cell line is a good cellular model for studying hematological malignancies characterized by *BCL2* over-expression. The cell line was originally derived from a patient with diffuse large B-cell lymphoma (DLBCL), harboring the t(14;18) translocation [26]. The pathogenesis of t(14;18) precludes the activation of the *BCL2* gene due to its juxtaposition under the regulatory influence of the *IGH* locus. In this study, we examined the nuclear organization of the loci involved in this rearrangement in the Pfeiffer cell line to better understand the mechanisms of transcriptional activation in relation to genome organization when a translocation does not result in the generation of a chimeric protein but in the activation of a proto-oncogene.

Our observations demonstrate that, in the Pfeiffer cell line, the chromosome territories contributing to the t(14;18) translocation do not show dramatic changes in their nuclear positioning compared to healthy lymphocytes. However, when observing the individual genes involved in the translocation, signals specific to the *BCL2::IGH* fusion indicate that the translocation may take the nuclear positioning of either of the wild-type genes. Previous reports on the nuclear positioning of translocation derivatives have documented various scenarios. For instance, the fusion gene *EWSR1::FLI1* resulting from t(11;22) in Ewing sarcoma has been reported to take an intermediate position in the cell nucleus when compared with wild-type *EWSR1* and *FLI1* [27]. Similarly, the translocation t(7;12)(q36,p13) in childhood acute myeloid leukemia results in the repositioning of the translocated *MNX1* gene to a more internal portion of the cell nucleus, driven by its translocation partner *ETV6* [28]. On the other hand, the Philadelphia chromosome resulting from t(9;22) in chronic myeloid leukemia does not change nuclear positioning compared to wild-type *BCR* and *ABL* since both genes occupy similar regions in the cell nucleus [29].

In this study, not only did we observe the positioning of translocation genes in the context of the cell nucleus, but we also looked at their positioning in relation to their chromosome territories. We noticed that *BCL2* was looping out of the chromosome territory only when translocated, hence protruding from the der(14) territory, in a relatively small percentage of nuclei. In absence of the translocation, we observed that *BCL2* remained within the chromosome 18 territory in normal lymphocytes, with no exception. This discrepancy may be due to a lack of *BCL2* expression in normal lymphocytes. Our observations strongly suggest that chromosome looping may be essential for *BCL2* expression in t(14;18) lymphoma, where the *BCL2* locus happens to be juxtaposed to the *IGH* promoter/enhancer on der(14). It remains to be clarified whether the new position of *BCL2* on der(14) requires looping in order to initiate transcription simply due to the usual process that would occur for *IGH* wild-type or whether *BCL2* looping is an essential process also in those rare cases where *BCL2* expression is observed in absence of the t(14;18) translocation. Further studies on a broader range of lymphoma cell lines will aim at elucidating this aspect. It has to be noted that chromosome looping occurs only in a proportion of nuclei in the Pfeiffer cell line. This may signify that *BCL2* is not transcribed simultaneously in all cells carrying the translocation at any given time. The phenomenon of chromosome looping is well documented and has been shown to be fundamental for the transcriptional up-regulation of genes [30,31,32]. The development of techniques of chromatin conformation capture (Hi-C) to study interactions within the genome has allowed the creation of a genome-wide catalog of chromatin loops [33,34,35]. This report provides further insight on the mechanism of alterations in genome organization in a disease situation caused by an acquired chromosomal rearrangement.

The long-term presence of t(14;18) in circulating blood has been documented in healthy individuals [36,37]. Given the physiological lifespan of naïve B-cells, the persistence of t(14;18) clones for several years could be attributed to a sustained expression of the *BCL2* gene to prevent programmed cell death. However, exposure to specific pathogens (e.g., hepatitis C virus) has been associated with the presence of long-lived memory B-cells [36,38]. Nevertheless, the occurrence of the translocation may represent a pre-lymphoma state, which requires additional mutational events to fully manifest the malignancy. At the molecular level, no changes have been observed between *BCL2::IGH* fusion junctions between patients with lymphoma and healthy individuals harboring t(14;18). Specific clones may take over with distinct advantageous characteristics, such as a higher expression of *BCL2*. It could be speculated that a reorganization of nuclear positioning may facilitate the transcriptional landscape required for the clones to propagate. In this context, one could also speculate that the changes seen at the level of genome organization and here described in the Pfeiffer cell line may not be related to the t(14;18) rearrangement itself but to the effect of some other mutations that changed the three-dimensional organization of the nucleus as a whole. Whilst this is plausible, there may also be changes to nuclear structures such as the lamina that would also impact genome organization with consequences on gene expression and cellular health [18,39,40]. Current genome editing technologies already make it possible to generate specific chromosomal rearrangements in a targeted manner. Future studies aimed at generating an in vitro model of t(14;18) lymphoma will be instrumental in defining the impact of this translocation on genome organization in this disease.

The relevance of genome organization studies through the analysis of chromosome territories and gene positioning has a potential value in cancer diagnosis and as a predictor of clinical outcome [41,42]. However, studies on lymphoma have been, so far, rather limited [43]. Previous studies have investigated the spatial organization of *BCL2* alleles in cancer pathologies such as cervical squamous epithelium neoplasia and have observed an unequal allelic *BCL2* gene positioning, speculating that transcriptional *BCL2* activation is associated with *BCL2* relocation towards the nuclear periphery [44]. The unravelling of 3D genome architecture is an emerging field in cell biology with implications for the understanding of mechanisms of disease development and progression, including cancer. With the advancement of genome editing technologies and a better understanding of 3D genome interactions, the future of biomedicine may be shaped towards finding ways to manipulate 3D nuclear architecture to restore health [45].

## 4. Materials and Methods

### 4.1. Cell Culture

The Pfeiffer cell line (CRL2632^TM^) was purchased from the American Tissue Culture Collection (ATCC, LGC standards, Teddington, UK) and was cultured in RMPI-1640, 10% fetal bovine serum (FBS), and 1% penicillin/streptomycin and incubated at 37 °C, 5% CO_2_, according to the instructions from the supplier.

### 4.2. Expression Analysis of Publicly Available Data

Expression of the *BCL2* gene in different lymphoma cell lines from the Cancer Cell Line Encyclopedia (CCLE) was retrieved from RNA sequencing data from the Expression Public 23Q4 project via the DepMap Portal accessible online at https://depmap.org/portal/ accessed on 15 December 2023. RNA sequencing data for the expression of *BCL2* in whole blood of healthy individuals were extracted from GTEx via the Xena platform [46]. Microarray expression data from patients with diffuse lymphoma were downloaded from GSE16131 [24]; the differential expression of *BCL2* was computed in the R environment (The R Foundation for statistical Computing, Vienna, Austria-version 4.3.2; Rstudio software, Boston, MA, USA-version 2023.12.0+369).

### 4.3. Control Samples

Archival chromosome and nuclei fixed suspensions from whole peripheral blood samples of healthy volunteers were obtained as part of previous studies [22]. Samples from four volunteers (two males and two females) aged between 20 and 30 years old were used. Ethical approval was obtained from the Ethics Committee of Brunel University London (Ethical approval no: 16516-TISS-Apr/2019-18741-2). Control cDNA from human fibroblasts used for reverse transcription (RT) PCR was obtained as part of previous studies [47]. Another cDNA used as a positive control in the RT-PCR experiments was obtained from the T-47D cell line, purchased from the American Tissue Culture Collection (ATCC).

### 4.4. Reverse Transcription (RT) PCR

Reverse transcription PCR was performed as previously described [22]. The primers used in this study are shown in Table 1.

### 4.5. Fluorescence In Situ Hybridization (FISH) on Metaphase Chromosomes and Interphase Nuclei

Fluorescence in situ hybridization experiments were performed as previously described [22]. The FISH probes used in this study are shown in Table 2.

Microscope images were captured and analyzed using the fluorescence microscope Zeiss Axioplan2 equipped with a Sensys cooled CCD camera (Zeiss, Oberkoche, Germany).

### 4.6. Nuclear Localization Analysis

The radial nuclear localization (RNL) of the PAC/BAC probes was obtained using a method previously described [22]. Briefly, a large number of hybridized nuclei were recorded, and each hybridization signal was radially localized by assigning a value between 0 and 1, with 0 being the value of the signals positioned in the center of the nucleus and 1 the peripheral edge of the nucleus. The data obtained from the analysis of at least 300 nuclei were analyzed using the software Prism v. 8.0 (GraphPad Software, San Diego, CA, USA).

Chromosome and gene positioning in interphase nuclei using erosion analysis was performed using 50+ images of flattened nuclei taken for each of the FISH probes used. The bespoke script delineates the nucleus using the DAPI signal; it then erodes in from the edge of each nucleus to the interior, constructing five shells of equal areas. The signal intensity of each FISH signal and the DAPI are measured in each shell (1–5). In order to normalize the data and extrapolate them to a 3D situation, the FISH gene measurement is divided by the DAPI signal measurement in each shell [25].

### 4.7. Statistical Analysis

Statistical analyses were performed with the software Prism v. 8.0 (GraphPad Software, San Diego, CA, USA) and Microsoft Excel v. 16.54. Statistical differences were determined by Student’s *t* test. The level of significance between groups was set to *p* < 0.05 (*), *p* < 0.01 (**), *p* < 0.001 (***), and *p* < 0.0001 (***).

## Figures and Tables

**Table 1:** Bcl-2 and β-actin primer sequences.

Primer Name	Sequence
β-actin Forward	5′-AAGAGAGGCATCCTCACCCT-3′
β-actin Reverse	5′-TACATGGCTGGGGTGTTGAA-3′
Bcl-2 Forward	5′-ACGGAGGCTGGGATGCCTTTG-3′
Bcl-2 Reverse	5‘-ACACGAAGCGGTGCTTGGCA-3′

**Table 2:** FISH probes used in this study.

Probe Name	Vector Type	Length, bp	Locus	Position	Labelling
RP5-998D24	PAC	165,821	*IGH*	14q32	Digoxigenin-FITC
RP11-299P2	BAC	146,688	*BCL2*	18q21	Biotin-Texas Red
WCP14 *	Paint			Chr 14	Biotin-Texas Red
WCP18 *	Paint			Chr 18	FITC

* From Cambio, Cambridge, UK.

## Data Availability

The data presented in this study are available on reasonable request from the corresponding author. The expression data analyzed here is available from: the Expression Public 23Q4 project via the DepMap Portal accessible online at https://depmap.org/portal/ (accessed on 15 November 2023); from GTEx via the Xena platform [47] (accessed on 6 February 2024); from GSE16131 [24] (accessed on 17 October 2023).
